# Metabolome in schizophrenia and other psychotic disorders: a general population-based study

**DOI:** 10.1186/gm233

**Published:** 2011-03-23

**Authors:** Matej Orešič, Jing Tang, Tuulikki Seppänen-Laakso, Ismo Mattila, Suoma E Saarni, Samuli I Saarni, Jouko Lönnqvist, Marko Sysi-Aho, Tuulia Hyötyläinen, Jonna Perälä, Jaana Suvisaari

**Affiliations:** 1 VTT Technical Research Centre of Finland, Tietotie 2, PO Box 1000, FI-02044 VTT, Espoo, Finland; 2 National Institute for Health and Welfare, Lintulahdenkuja 4, PO Box 30, FI-00271, Helsinki, Finland; 3 Department of Psychiatry, Helsinki University Central Hospital, Välskärinkatu 12, PO Box 590, FIN-00029 HUCH, Helsinki, Finland

## Abstract

**Background:**

Persons with schizophrenia and other psychotic disorders have a high prevalence of obesity, impaired glucose tolerance, and lipid abnormalities, particularly hypertriglyceridemia and low high-density lipoprotein. More detailed molecular information on the metabolic abnormalities may reveal clues about the pathophysiology of these changes, as well as about disease specificity.

**Methods:**

We applied comprehensive metabolomics in serum samples from a general population-based study in Finland. The study included all persons with DSM-IV primary psychotic disorder (schizophrenia, *n *= 45; other non-affective psychosis (ONAP), *n *= 57; affective psychosis, *n *= 37) and controls matched by age, sex, and region of residence. Two analytical platforms for metabolomics were applied to all serum samples: a global lipidomics platform based on ultra-performance liquid chromatography coupled to mass spectrometry, which covers molecular lipids such as phospholipids and neutral lipids; and a platform for small polar metabolites based on two-dimensional gas chromatography coupled to time-of-flight mass spectrometry (GC × GC-TOFMS).

**Results:**

Compared with their matched controls, persons with schizophrenia had significantly higher metabolite levels in six lipid clusters containing mainly saturated triglycerides, and in two small-molecule clusters containing, among other metabolites, (1) branched chain amino acids, phenylalanine and tyrosine, and (2) proline, glutamic, lactic and pyruvic acids. Among these, serum glutamic acid was elevated in all psychoses (*P *= 0.0020) compared to controls, while proline upregulation (*P *= 0.000023) was specific to schizophrenia. After adjusting for medication and metabolic comorbidity in linear mixed models, schizophrenia remained independently associated with higher levels in seven of these eight clusters (*P *< 0.05 in each cluster). The metabolic abnormalities were less pronounced in persons with ONAP or affective psychosis.

**Conclusions:**

Our findings suggest that specific metabolic abnormalities related to glucoregulatory processes and proline metabolism are specifically associated with schizophrenia and reflect two different disease-related pathways. Metabolomics, which is sensitive to both genetic and environmental variation, may become a powerful tool in psychiatric research to investigate disease susceptibility, clinical course, and treatment response.

## Background

Psychotic disorders are among the most severe and impairing medical diseases [1]. Schizophrenia is the most common of them, with a lifetime prevalence of 1% in a general population [2]. The current view is that schizophrenia is a developmental disorder caused by a combination of genetic vulnerability, early environmental insults, subtle developmental and cognitive impairments, and later influences such as social adversity and drug abuse [3], with heritability of about 80% [4,5]. The *Diagnostic and Statistical Manual of Mental Disorders *(DSM)-IV divides primary psychotic disorders into nine different diagnoses based on symptom patterns, clinical course and outcome, although it is unclear whether this has any etiological justification. Nevertheless, while there is overlap in genetic vulnerability between different psychotic disorders, like schizophrenia and bipolar I disorder, they also have non-shared genetic and environmental risk factors [5,6]. Given the multi-factorial complexity of psychotic disorders [7], identification of molecular markers sensitive to the underlying pathogenic factors of specific diseases would be of high relevance, not only to assist in their early detection and diagnosis, but also to subsequently facilitate disease monitoring and treatment responses.

Metabolomics is a discipline dedicated to the global study of small molecules (that is, metabolites) in cells, tissues, and biofluids. Concentration changes of specific groups of circulating metabolites may be sensitive to pathogenically relevant factors, such as genetic variation, diet, age, or gut microbiota [8-12]. Over the past years, technologies have been developed that allow comprehensive and quantitative investigation of a multitude of different metabolites [13]. The study of high-dimensional chemical signatures as obtained by metabolomics may therefore be a powerful tool for characterization of complex phenotypes affected by both genetic and environmental factors [14]. Previous metabolomic studies in schizophrenia and related psychoses have highlighted the importance of glucoregulatory processes [15,16] and tryptophan metabolism [17] in psychosis, and lipidomics approaches have identified specific drug-response profiles for three commonly used atypical antipsychotics [18]. However, no metabolomics studies have so far been conducted to discriminate between different groups of psychotic disorders.

Here we sought to determine the serum metabolic profiles associated with different psychotic disorders, clustered into three main categories: schizophrenia, affective psychoses, and other non-affective psychoses (ONAP). A metabolomics approach with broad analytical coverage was applied to serum samples from a well characterized population cohort [2]. We investigated dependencies of the three different diagnostic groups on specific metabolic profiles in the context of metabolic comorbidity, antipsychotic medication as well as other lifestyle variables.

## Materials and methods

### Study population

The subjects are from the Health 2000 survey, which is based on a nationally representative sample of 8,028 people aged 30 years or over from Finland [19]. A two-stage stratified cluster sampling procedure was used. The field work took place between September 2000 and June 2001, and consisted of a home interview and a health examination at the local health center, or a condensed interview and health examination of non-respondents at home. In addition, register information was gathered on the whole sample. The Health 2000 study and the accompanying Psychoses in Finland study were approved by the Ethics Committees of the National Public Health Institute (since 2009 the National Institute for Health and Welfare) and the Hospital District of Helsinki and Uusimaa, and participants gave written informed consent [19]. The response rate in the survey, 93%, was exceptionally high compared with other recent surveys.

In the Psychoses in Finland study, we screened people with possible psychotic disorders from the Health 2000 study sample and interviewed them using the Research Version of the Structured Clinical Interview for DSM-IV (SCID-I) [20]. People were invited to participate in the SCID interview if they reported having been diagnosed with a psychotic disorder, received a diagnosis of a possible or definite psychotic disorder from the physician conducting the health examination, or reported possible psychotic or manic symptoms in the Composite International Diagnostic Interview [21] conducted as part of the health examination. A register-based screen was also used, including hospital treatment for a diagnosis of any psychotic disorder, reimbursement for antipsychotic medication, receipt of a disability pension because of a psychotic disorder, or use of mood-stabilizing medication without a diagnosis of any relevant medical condition, such as epilepsy [2].

Of the screen-positive people, 63.4% participated in the SCID interview. We diagnosed those who did not participate in the interview using hospital and outpatient case notes from psychiatric and primary care units. Case notes for those who participated in the interview were also collected. Final DSM-IV-based diagnoses were made by JS, JP, and SIS using all available information. Kappa values between the raters ranged from 0.74 to 0.97 for different psychotic disorders [2].

In this study, lifetime diagnoses of psychotic disorders were grouped into schizophrenia, ONAPs (schizophreniform disorder, schizoaffective disorder, delusional disorder, brief psychotic disorder, psychotic disorder not otherwise specified), and affective psychosis (major depressive disorder with psychotic features and bipolar I disorder). The final study sample comprised 45 subjects with schizophrenia (19 men), 57 with ONAP (20 men), and 37 with affective psychosis (23 men) for whom serum samples were available. There were more women than men in the schizophrenia and ONAP groups, which reflects the gender distribution in the Finnish general population aged 30 years and over and the higher prevalence of schizoaffective disorder in women than in men [2]. An equal number of controls, matched for age, sex, and region of residence, was selected for each group (Table 1). Most of the antipsychotics used by patients were first-generation antipsychotics (Table 1). A total of 12 subjects in the sample used second-generation antipsychotics, of whom 7 used risperidone, 4 clozapine, and one olanzapine. There were 54 subjects who used first-generation antipsychotics, of which the most commonly used were perphenazine (22 users) and thioridazine (16 users).

**Table 1:** Demographic characteristics and mean values and χ^2 ^tests^a ^of variables related to metabolic comorbidity for persons with psychotic disorders and their matched controls

	Schizophrenia			Other non-affective psychosis			Affective psychosis		
			
Variable	Cases	Controls	*P*-value	Cases	Controls	*P*-value	Cases	Controls	*P*-value
Age (years)	53.7 (12.9)	53.7 (12.9)	NS	54.7 (14.3)	54.7 (14.3)	NS	54.7 (14.8)	54.7 (14.9)	NS
Sex									
Male	19	19	NS	20	20	NS	23	23	NS
Female	26	26		37	37		14	14	
Antipsychotic medication use									
Current	34 (75.6%)	0 (0%)	<0.001	24 (42.1%)	0 (0%)	<0.001	8 (21.6%)	0 (0%)	0.003
Atypical antipsychotics	8 (17.0%)	0 (0%)		4 (7.0%)	0 (0%)		0 (0%)	0 (0%)	
Lifetime	44 (97.8%)	NA		50 (87.7%)	NA		34 (91.9%)	NA	
Type 2 diabetes	11 (24.4%)	3 (6.7%)	0.020	8 (14.0%)	4 (7.0%)	NS	0 (0%)	3 (8.1%)	NS
Metabolic syndrome	19 (42.2%)	13 (28.9%)	NS	25 (43.9%)	15 (26.3%)	0.048	10 (27.0%)	11 (29.7%)	NS
Metabolic comorbidity^b^	22 (48.9%)	15 (33.3%)	NS	33 (57.9%)	21 (36.8%)	0.024	14 (37.8%)	14 (37.8%)	NS
Daily smoking	20 (44.4%)	12 (26.7%)	NS	17 (29.8%)	15 (26.3%)	NS	10 (27.0%)	9 (24.3%)	NS
Daily use of vegetables	20 (45.5%)^d^	32 (71.1%)	0.014	23 (41.1%)^d^	35 (61.4%)	0.031	19 (51.4%)	20 (54.1%)	NS
Daily use of milk with high fat %	20 (46.5%)^e^	16 (36.4%)	NS	21 (37.5%)^d^	16 (28.6%)^d^	NS	15 (40.5%)	12 (32.4%)	NS
Daily use of vegetable oils	27 (62.8%)^e^	31 (68.9%)	NS	35 (61.4%)^d^	42 (75.0%)	NS	25 (67.6%)	22 (59.5%)	NS
Daily use of cheese with high fat content	8 (19.1%)^f^	33.3% (15)	NS	16 (28.6%)^d^	14 (25.0%)^d^	NS	9 (24.3%)	16 (43.2%)	NS
Body mass index (kg/m^2^)	28.4 (5.8)	26.1 (3.3)	NS	28.8 (6.2)	26.6 (3.9)	NS	27.5 (3.7)	26.4 (4.1)	NS
Systolic blood pressure	128.4 (20.1)	134.3 (20.7)	NS	131.6 (17.8)	140.8 (25.4)	NS	128.1 (18.8)	135.4 (20.1)	NS
Diastolic blood pressure	79.8 (10.7)	80.5 (12.0)	NS	82.3 (10.5)	82.7 (10.0)	NS	79.9 (10.4)	81.5 (9.9)	NS
Plasma glucose (mg/dl)	109.9 (31.9)	97.2 (12.3)	0.016	106.5 (42.5)	101.6 (15.0)	NS	97.0 (12.0)	100.2 (14.6)	NS
Serum cotinine (μg/l)	216.2 (317.2)	96.8 (207.1)	0.030	151.4 (249.4)	121.2 (253.5)	NS	124.5 (234.2)	150.4 (284.6)	NS
Serum total cholesterol (mg/dl)^c^	226.0 (50.0)	229.7 (37.9)	NS	232.3 (41.6)	224.7 (39.6)	NS	230.0 (40.0)	237.1 (37.0)	NS
Serum HDL cholesterol (mg/dl)	45.3 (13.5)	54.5 (14.5)	0.003	49.7 (14.3)	51.6 (14.6)	NS	45.0 (13.0)	50.5 (16.7)	NS
Serum triglycerides (mg/dl)	197.4 (130.2)	120.6 (55.2)	0.006	156.5 (112.6)	125.9 (81.2)	0.044	151.4 (97.2)	144.5 (85.0)	NS
Serum insulin (μIU/ml)	16.6 (19.6)	7.6 (5.4)	<0.001	11.9 (12.4)	8.4 (5.8)	NS	9.6 (6.1)	9.3 (7.2)	NS
HOMA-IR	4.81 (6.98)	1.84 (1.28)	<0.001	4.19 (10.99)	2.17 (1.74)	NS	2.33 (1.53)	2.42 (2.25)	NS
Fasting time (hours)	6.40 (4.17)	7.13 (3.89)	NS	9.29 (5.98)	7.87 (4.23)	NS	6.43 (3.98)	8.37 (5.06)	NS
Waist circumference (cm)	98.8 (15.1)	89.5 (11.7)	0.003	97.4 (16.4)	90.8 (12.4)	0.037	97.4 (12.2)	93.1 (12.6)	NS
C-reactive protein (mg/l)	2.5 (2.8)	1.7 (3.3)	0.004	3.7 (4.9)	2.2 (4.3)	0.017	1.9 (2.9)	1.0 (1.4)	NS
BDI score	13.5 (10.9)	5.7 (4.4)	<0.001	14.9 (12.3)	6.5 (6.1)	<0.001	11.1 ( 9.3)	6.0 (5.6)	0.029

Standard deviations for continuous variables and percentages for categorical variables are reported in parentheses. ^a^*P*-values from χ^2 ^tests for categorical and Mann-Whitney *U *tests for continuous variables. ^b^Metabolic comorbidity: type 2 diabetes, metabolic syndrome, or obesity (BMI ≥30). ^c^To convert cholesterol to mmol/l, multiply values by 0.0259; to convert triglycerides to mmol/l, multiply value by 0.0113; to convert glucose to mmol/l, multiply values by 0.0555; and to convert insulin to pmol/l, multiply values by 6.945. ^d^Information missing from one participant. ^e^Information missing from two participants. ^f^Information missing from three participants. Abbreviations: BDI, Beck Depression Inventory [26]; BMI, body mass index; HOMA-IR, homeostasis model assessment index; NA, not applicable (information on lifetime antipsychotic exposure was not available from controls); NS, not statistically significant.

### Blood samples

Participants were asked to fast a minimum of 4 hours before the examination. Subjects with antidiabetic medication were allowed to take their medication and meals at the time they would usually take them (the number of such subjects was three in the schizophrenia group and two in their controls, six in the ONAP group and two in their controls, none in the affective psychosis group and one in their controls). Blood samples were taken at the beginning of the health examination or home health examination. Serum samples were separated, aliquoted and subsequently stored at -70°C (-94°F).

### Biochemical measures

Total, high-density lipoprotein (HDL), and low-density lipoprotein (LDL) cholesterol, triglycerides and glucose were measured with an AU400 analyzer (Olympus, Japan). The inter-assay coefficient of variation for glucose (Olympus System reagent, O'Callaghan's Mills, Co. Clare, Ireland), triglycerides (Olympus System reagent), total cholesterol (Olympus System reagent), HDL cholesterol (HDL-C Plus, Roche Diagnostics, Mannheim, Germany), and LDL cholesterol (LDL-C Plus, Roche Diagnostics) was 2.3%, 3.2%, 2.2%, 5.3%, and 5.7%, respectively. Serum insulin concentrations were determined with an IMx analyzer (Abbott Laboratories, Abbott Park, IL, USA) by microparticle enzyme immunoassay. C-reactive protein (CRP) was determined using an ultra-sensitive immunoturbidometric test (Orion Diagnostica, Espoo, Finland) on an Optima analyzer (Thermo Electron Corporation, Vantaa, Finland). The inter-assay coefficient of variation of both insulin and CRP assays was 4.5%. The cotinine concentration was determined from serum using a radioimmunoassay methodology (Nicotine Metabolite Double Antibody kit, Diagnostic Products Corporation, Los Angeles, CA, USA). The inter-assay coefficient of variation was 12.3%.

### Other measures

Blood pressure was measured after a 5-minute rest twice from the right upper arm with the person sitting. Values used here are average values from the measurements. Weight was measured during bioimpedance measurement. Waist circumference was measured while standing, midway between the lowest rib and the iliac crest, after a modest expiration [22].

Type 2 diabetes was diagnosed according to the World Health Organization 1999 criteria [23], combining information from several sources: self-reported diagnosis of type 2 diabetes that was further confirmed in the clinical examination; antidiabetic medication use based on self-report or health care registers; or fasting plasma glucose ≥126 mg/dl (7.0 mmol/l) or nonfasting glucose ≥200 mg/dl (11.1 mmol/l) [24]. Metabolic syndrome was diagnosed using the National Cholesterol Education Program's Adult Treatment Panel III (ATPIII) criteria [25].

The quantity of alcohol consumption was investigated by asking the respondents to report their average weekly consumption during the past month, separately for each type of alcoholic beverage. The answers were converted into grams of alcohol per week. Daily smoking was self-reported and was defined as having smoked at least 100 cigarettes, having smoked for at least 1 year, and having smoked during the day of the interview or the day before. Standard, validated diet-related questions were used to assess the habitual use of vegetable oils versus butter, use of and fat content in milk products, and daily use of raw vegetables [22].

The Beck Depression Inventory (BDI-21) [26] was used to assess current depressive symptoms.

### Lipidomic analysis by ultra-performance liquid chromatography coupled to mass spectrometry

EDTA-blood samples (10 ml) were centrifuged at 3,200 rpm (1600 G) for 15 minutes at room temperature within 2 hours of blood sampling. Serum was separated and stored at -80°C. For lipidomics profiling, 10 μl aliquots of serum were used. The samples were mixed with 10 μl of 0.9% (0.15 M) sodium chloride in Eppendorf tubes, spiked with a standard mixture consisting of 10 lipids (0.2 μg/sample; PC(17:0/0:0), PC(17:0/17:0), PE(17:0/17:0), PG(17:0/17:0), Cer(d18:1/17:0), PS(17:0/17:0), PA(17:0/17:0), MG(17:0/0:0/0:0)[rac], DG(17:0/17:0/0:0)[rac], TG(17:0/17:0/17:0), where PC is phosphatidylcholine, PE is phosphatidylethanolamine, PG is phosphatidylglycerol, Cer is ceramide, PS is phosphatidylserine, PA is phosphatidic acid, MG is monoglyceride, DG is diglyceride, and TG is triglyceride) and extracted with 100 μl of chloroform/methanol (2:1). After vortexing (2 minutes) and standing (1 hour) the tubes were centrifuged at 10,000 rpm (7826 G) for 3 minutes and 60 μl of the lower organic phase was separated and spiked with a standard mixture containing three labeled lipids (0.1 μg/sample; PC(16:0/0:0-D_3_), PC(16:0/16:0-D_6_), TG(16:0/16:0/16:0-^13^C3)).

Lipid extracts were analyzed in a randomized order on a Waters Q-Tof Premier mass spectrometer combined with an Acquity UltraPerformance LC™ system (UPLC) (Waters Corporation, Milford, MA, USA). The column (at 50°C) was an Acquity UPLC™ BEH C18 1 × 50 mm with 1.7 μm particles. The solvent system included ultrapure water (1% 1 M NH_4_Ac, 0.1% HCOOH) and liquid chromatography/mass spectrometry (MS) grade acetonitrile/isopropanol (5:2, 1% 1 M NH_4_Ac, 0.1% HCOOH). The gradient started from 65% A/35% B, reached 100% B in 6 minutes and remained there for the next 7 minutes. There was a 5-minute re-equilibration step before the next run. The flow rate was 0.200 ml/minute and the injected amount 1.0 μl (Acquity Sample Organizer). Reserpine was used as the lock spray reference compound. The lipid profiling was carried out using ESI+ mode and the data were collected at a mass range of m/z 300 to 1,200 with a scan duration of 0.2 s.

The data were processed by using MZmine 2 software [27] and the lipid identification was based on an internal spectral library [28].

### Metabolomic analysis by two-dimensional gas chromatography coupled to time-of-flight MS

Each serum sample (30 μl) was spiked with an internal standard (7 μl 258 ppm labeled palmitic acid) and the mixture was then extracted with 400 μl of methanol. Labeled d-valine (10 μl, 37 ppm) was added to the extracts as a derivatization standard. After centrifugation the supernatant was evaporated to dryness and the original metabolites were then converted into their trimethylsilyl (TMS) and methoxime derivative(s) by two-step derivatization. First, 25 μl methoxyamine hydrochloride (MOX) reagent was added to the residue and the mixture was incubated for 60 minutes at 45°C. Next, 25 μl N-methy-N-(trimethylsilyl) trifluoroacetamide was added and the mixture was incubated for 60 minutes at 45°C. The derivatized samples were diluted 1:1 with hexane. Finally, a retention index standard mixture (n-alkanes) and an injection standard (4,4'-dibromooctafluorobiphenyl), both in pyridine, were added to the mixture.

For the analysis, a Leco Pegasus 4D GC × GC-TOFMS (two-dimensional gas chromatography coupled to time-of-flight MS) instrument (Leco Corp., St Joseph, MI, USA) equipped with a cryogenic modulator was used. The GC part of the instrument was an Agilent 6890N gas chromatograph (Agilent Technologies, Palo Alto, CA, USA) equipped with a split/splitless injector. For the injection, a pulsed splitless injection (0.5 μl) at 240°C was used, with pulse pressure of 55 psig for 1 minute. The first-dimension chromatographic column was a 10-m RTX-5 capillary column with an internal diameter of 0.18 mm and a stationary-phase film thickness of 0.20 μm, and the second-dimension chromatographic column was a 1.5-m BPX-50 capillary column with an internal diameter of 100 μm and a film thickness of 0.1 μm. A diphenyltetramethyldisilyl deactivated retention gap (3 m × 0.53 mm internal diameter) was used in the front of the first column. High-purity helium was used as the carrier gas at a constant pressure mode (39.6 psig). A 5-s separation time was used in the second dimension. The MS spectra was measured at 45 to 700 amu with 100 spectra per second. Pulsed splitless injection 0.5 μl at 240°C was used. The temperature program was as follows: the first-dimension column oven ramp began at 40°C with a 2-minute hold, after which the temperature was programmed to 295°C at a rate of 7°C/minute and then held at this temperature for 3 minutes; the second-dimension column temperature was maintained 20°C higher than the corresponding first-dimension column. The programming rate and hold times were the same for both columns.

### Cluster analysis

The data were scaled into zero mean and unit variance to obtain metabolite profiles comparable to each other. Bayesian model-based clustering was applied on the scaled data to group lipids with similar profiles across all samples. The analyses were performed using the MCLUST [29] method, implemented in R [30] as package 'mclust'. In MCLUST the observed data are viewed as a mixture of several clusters and each cluster comes from a unique probability density function. The number of clusters in the mixture, together with the cluster-specific parameters that constrain the probability distributions, will define a model that can then be compared to others. The clustering process selects the optimal model and determines the data partition accordingly. The number of clusters ranging from 4 to 15 and all available model families were considered in our study. Models were compared using the Bayesian information criterion, which is an approximation of the marginal likelihood. The best model is the one that gives the largest marginal likelihood of data, that is, the highest Bayesian information criterion value.

### Descriptive statistical analyses and linear mixed models

Differences between each diagnostic group and their matched controls in metabolic comorbidity, lifestyle-related factors, mood, and glucose and lipid measurements were compared using the χ^2 ^test for categorical variables and Mann-Whitney *U *test for continuous variables. One-way analysis of variance (ANOVA), implemented in Matlab (MathWorks, Natick, MA, USA), was applied to compare the average metabolite profiles in each metabolite cluster. Individual metabolite levels were visualized using the beanplots [31], implemented in the 'beanplot' R package [30]. Beanplot provides information on the mean metabolite level within each group, the density of the data-point distribution, as well as shows individual data points. The independent effects of diagnostic categories, current antipsychotic medication use, metabolic comorbidity (that is, type 2 diabetes, metabolic syndrome, and obesity (body mass index ≥30)), diet (use of vegetable oil versus butter, use of milk and cheese with high fat content, daily use of vegetables), and duration of fasting were analyzed using linear mixed models [32] that took the matching of case-control pairs into account. Because the matching was based on both sex and age, these were not included in the models as independent variables. This analysis was performed using PROC MIXED in SAS statistical software, version 9.1.3 (Cary, NC, USA). Logarithm transformations were applied to the metabolomics cluster values to improve normality.

### Partial correlation network analysis

Construction of the dependency network for selected variables was performed using undirected Gaussian graphical Markov networks that represent *q*-order partial correlations between variables, implemented in the R package 'qpgraph' [33] from the Bioconductor project [34]. In these networks missing edges denote zero partial correlations between pairs of variables, and thus imply the conditional independence relationships in the Gaussian case.

Structure learning of the Gaussian graphs corresponds to a statistical test such as *t*-test for the hypothesis that a given *q*-order partial correlation is zero. If all of such hypotheses of zero *q*-order partial correlations are rejected, then the two variables are joined by an edge. In practice, we tested the hypothesis by default with four equidistant *q*-values along the (1, 52) interval, namely *q *= 1, 13, 26 and 38. For each of the *q*-values, the test was repeated for each pair of variables by sampling 500 elements randomly selected from the subsets of the data that contain *q *variables. A missing edge is identified if the proportion of such tests where the null-hypothesis is not rejected - for example, the average non-rejection rate of the hypothesis - is above a certain threshold. A small average non-rejection rate therefore implies strong evidence of dependence. The resulting graph can thus be obtained by removing all the missing edges from the complete graph. Unlike Pearson correlation coefficients, use of partial correlation adjusts for the confounding effects and thus removes spurious associations to a large extent. The network was visualized using Cytoscape [35] and yED graphical editor [36].

### Diagnostic model

A logistic regression model implemented in R was applied to discriminate the 45 schizophrenia patients from the 94 other participants diagnosed with psychoses using four selected metabolic markers. In order to assess the best marker combination, 10,000 cross-validation runs were performed. In each run, 93 and 46 samples were selected at random as the training and test sets, respectively, and the best marker combination in the logistic regression model was selected using a stepwise algorithm using Akaike's information criterion [37]. The best model was then applied to the test set samples to calculate their predicted classes. The optimal marker combinations in each of the cross-validation runs, receiver operating characteristic (ROC) curves with area under the curve (AUC) statistics, odds-ratios and relative risks were recorded.

## Results

### Metabolomic analysis

Two analytical platforms for metabolomics were applied to all serum samples: a global lipidomics platform based on UPLC-MS, which covers molecular lipids such as phospholipids, sphingolipids, and neutral lipids; and a platform for small polar metabolites based on GC × GC-TOFMS covers small molecules such as amino acids, free fatty acids, keto-acids, various other organic acids, sterols, and sugars. Both platforms were recently described and applied in a large prospective study in type 1 diabetes [12]. The final dataset from each platform consisted of a list of metabolite peaks (identified or unidentified) and their concentrations, calculated using the platform-specific methods, across all samples. All metabolite peaks were included in the data analyses, including the unidentified ones. We reasoned that inclusion of complete data as obtained from the platform best represents the global metabolome, and the unidentified peaks may still be followed-up later on with *de novo *identification using additional experiments if deemed of interest.

### Associations of global metabolome with psychotic disorders

A total of 360 molecular lipids and 201 metabolites were measured, of which 170 and 155 were identified, respectively. Due to a high degree of co-regulation among the metabolites [38], one cannot assume that all the 562 measured metabolites are independent. The global metabolome was therefore first surveyed by clustering the data into a subset of clusters using the Bayesian model-based clustering [29]. Lipidomic platform data were decomposed into 13 clusters (LC1 to LC13) and the metabolomic data into 8 clusters (MC1 to MC8). Descriptions of each cluster and representative metabolites are provided in Table 2. As expected, the division of clusters to a large extent follows different metabolite functional or structural groups.

**Table 2:** Description of metabolite clusters obtained from lipidomic (LC) or metabolomics (MC) platforms

Cluster name	Cluster size	Description	Examples of metabolites	Significant predictors
LC1	112	Major phospholipids, such as PC, lysoPC, SM	lysoPC(16:0), PC(34:2), SM(d18:1/16:0)	None
LC2	48	Mainly PUFA-containing PCs	PC(16:1/22:6), PC(18:1/20:4)	None
LC3	11	PUFA-containing PCs and PEs	PE(16:0/22:6), PC(18:0/22:6)	None
LC4	15	Short chain saturated TGs	TG(44:0), TG(16:0/16:0/16:0)	Schizophrenia (↑, *t *= 3.72, *P *= 0.0003), metabolic comorbidity (↑, *t *= 6.00, *P *< 0.0001), daily use of cheese with high fat content (↑, *t *= 2.45, *P *= 0.016)
LC5	31	Mainly unidentified, includes short odd-chain TG	TG(43:0)	Schizophrenia (↑, *t *= 2.03, *P *= 0.045), metabolic comorbidity (↑, *t *= 3.09, *P *= 0.003)
LC6	21	Odd-chain TGs, mainly saturated or monounsaturated	TG(47:0), TG(47:1)	Schizophrenia (↑, *t *= 2.27, *P *= 0.025), metabolic comorbidity (↑, *t *= 4.14, *P *< 0.0001), daily use of cheese with high fat content (↑, *t *= 2.29, *P *= 0.024)
LC7	20	Mainly odd-chain TGs, longer fatty acids than LC5 and LC6	TG(15:0/16:0/18:1), TG(51:2), TG(50:2), TG(16:0/16:0/18:1)	Schizophrenia (↑, *t *= 3.20, *P *= 0.002), metabolic comorbidity (↑, *t *= 7.99, *P *< 0.0001), daily use of cheese with high fat content (↑, *t *= 2.06, *P *= 0.042)
LC8	34	Medium- and long-chain TGs	TG(18:1/16:0/18:1), TG(18:1/16:0/18:2), TG(18:1/18:1/18:1), TG(18:1/18:2/18:1)	Schizophrenia (↑, *t *= 3.08, *P *= 0.003), metabolic comorbidity (↑, *t *= 7.04, *P *< 0.0001)
LC9	17	Longer-chain, SFA- and MUFA-containing TGs	TG(18:0/18:0/18:1), TG(18:1/18:0/18:1), TG(18:0/18:0/16:0)	Schizophrenia (↑, *t *= 4.23, *P *< 0.0001), metabolic comorbidity (↑, *t *= 6.72, *P *< 0.0001), daily use of cheese with high fat content (↑, *t *= 2.93, *P *= 0.004), fasting time (↓, *t *= -1.98, *P *= 0.050)
LC10	21	PUFA containing long-chain TGs	TG(16:0/18:1/22:6), TG(56:8), TG(16:0/16:1/22:6), TG(58:9)	Metabolic comorbidity (↑, *t *= 5.28, *P *< 0.0001)
LC11	9	Unknown lipids		Use of vegetable oils (↓, *t *= -2.61, *P *= 0.010), fasting time (↓, *t *= -2.06, *P *= 0.041)
LC12	7	Unknown lipids		Use of vegetable oils (↓, *t *= -2.24, *P *= 0.027)
LC13	5	Unknown lipids		None
MC1	34	Sugars, sugar acids, urea metabolites	Allonic acid, myo-inositol, glycopyranose, urea	Metabolic comorbidity (↑, *t *= 3.10, *P *= 0.002), fasting time (↓, *t *= -2.46, *P *= 0.015)
MC2	18	Ketone bodies, free fatty acids	Acetoacetic acid, beta-hydroxybutyric acid, stearic acid, oleic acid	Schizophrenia (↓, *t *= -2.68, *P *= 0.009), affective psychosis (↓, *t *= -2.79, *P *= 0.006), antipsychotic use (↑, *t *= 2.45, *P *= 0.016)
MC3	10	Branched chain amino acids and other amino acids	Isoleucine, phenylalanine, tyrosine, ornithine, serine, methionine, threonine	Schizophrenia (↑, *t *= 2.03, *P *= 0.045)
MC4	53	Energy metabolites, various organic acids	Hippuric acid, glycine, succinic acid, fumaric acid, alpha-linolenic acid, adipic acid	Antipsychotic use (↓, *t *= -2.16, *P *= 0.033)
MC5	38	Amino acids, organic acids	Proline, glutamic acid, alpha-ketoglutaric acid, pyruvic acid, alanine, lactic acid, alpha-hydroxybutyrate	Schizophrenia (↑, *t *= 2.35, *P *= 0.020), metabolic comorbidity (↑, *t *= 5.19, *P *< 0.0001), fasting time (↓, *t *= -2.34, *P *= 0.021)
MC6	25	Various organic acids	Arachidonic acid, aminomalonic acid, citric acid	None
MC7	17	Mainly unidentified carboxylic acids and alcohols	Beta-sitosterol	None
MC8	6	Lipid metabolites	2-Monopalmitin	None

The rightmost column shows the results from linear mixed models, with diagnostic categories, current antipsychotic medication use, metabolic comorbidity (that is, type 2 diabetes, metabolic syndrome, and obesity (body mass index ≥30)), diet (use of vegetable oil versus butter, use of milk and cheese with high fat content, daily use of vegetables) and hours of fasting. Abbreviations: lysoPC, lysophosphatidylcholine; MUFA, monounsaturated fatty acid; PC, phosphatidylcholine, PE, phosphatidylethanolamine; PUFA, polyunsaturated fatty acid; SFA, saturated fatty acid; SM, sphingomyelin; TG, triglyceride.

**Figure 1:**
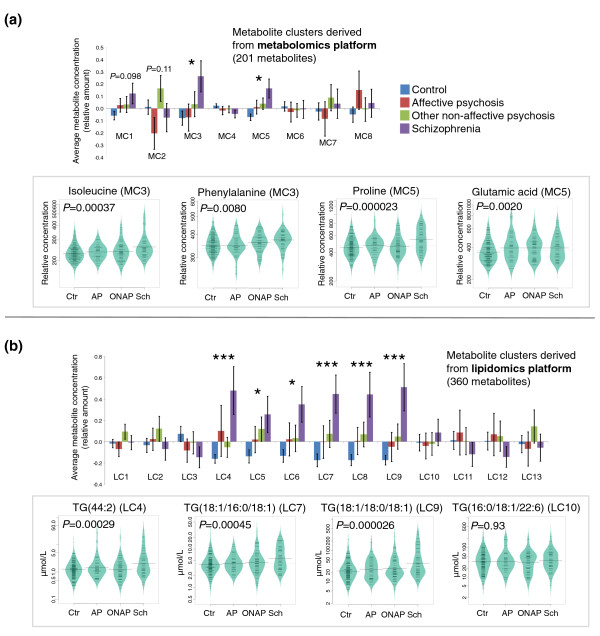
**Mean metabolite levels within each cluster across the three diagnostic groups and the controls**. Data were obtained from the **(a) **metabolomics (GC × GC-TOFMS) and **(b) **lipidomics (UPLC-MS) platforms. Error bars show standard error of the mean (*P < 0.05, ***P < 0.001). For each platform, profiles of selected representative metabolites from different clusters are also shown. The order of fatty acids in the reported triglycerides was not uniquely determined. The metabolite levels are shown as beanplots [31], which provide information on the mean level (solid line), individual data points (short lines), and the density of the distribution. The concentration scale in beanplots is logarithmic. Abbreviations: Ctr, control; AP, affective psychoses; ONAP, other non-affective psychoses; Sch, schizophrenia.

As shown in Figure 1, several of the clusters had different average metabolite profiles across the four diagnostic groups, with the control groups pooled into one in this part of the analysis. The average profiles of the lipid clusters LC4 to LC9, which predominantly contained TGs, were most elevated in the schizophrenia group, although the ONAP and affective psychosis groups also tended to have higher TGs compared to controls. The differences were most pronounced for TGs containing more saturated fatty acids, while the cluster containing TGs with polyunsaturated fatty acids (LC10) did not differ between the groups. Two small-molecule clusters were upregulated in schizophrenia, MC3 and MC5, containing branched chain amino acids (BCAAs) and other amino acids, including proline, phenylalanine and glutamic acid. A cluster containing various sugar molecules, MC1, displayed a similar pattern to those of MC3 and MC5, but at a marginal significance level. Cluster MC2, which contained ketone bodies, keto-acids as well as specific free fatty acids, had a distinct pattern that separated the (high level) ONAP and the (low level) affective psychosis groups.

### Metabolic comorbidity, antipsychotic medication use, and other lifestyle

It is known that psychoses are associated with metabolic comorbidities [2] and that the lipid profiles as measured by lipidomics in schizophrenic patients are greatly affected by the use of specific antipsychotic medication [18]. In order to assess the disease-specificity of the observed metabolic changes, the linear mixed effects models were applied on individual metabolite clusters, which included the three diagnostic categories, metabolic comorbidity, current antipsychotic medication, and diet as well as fasting time as explanatory variables (Table 2).

The TG-containing lipid clusters (LC4 to LC10) all associated with metabolic comorbidity, but most of them were also independently and positively associated with schizophrenia. Diet-related factors also affected most of them. Surprisingly, none of the lipid clusters associated with antipsychotic medication use after taking diagnoses, metabolic comorbidity and diet into account. Metabolite cluster MC5 was positively associated with both schizophrenia and metabolic comorbidity, while one (MC3) was associated only with schizophrenia. The only cluster associated with psychoses other than schizophrenia was MC2, which was negatively associated with schizophrenia and affective psychosis. One cluster, MC4, containing various organic acids and energy metabolites, was specifically negatively associated with antipsychotic use.

The observed associations of lipid and metabolic clusters with schizophrenia remained significant in most clusters if patients diagnosed with type 2 diabetes and their controls were excluded from the analysis (Additional file 1).

### Dependency analysis

**Figure 2:**
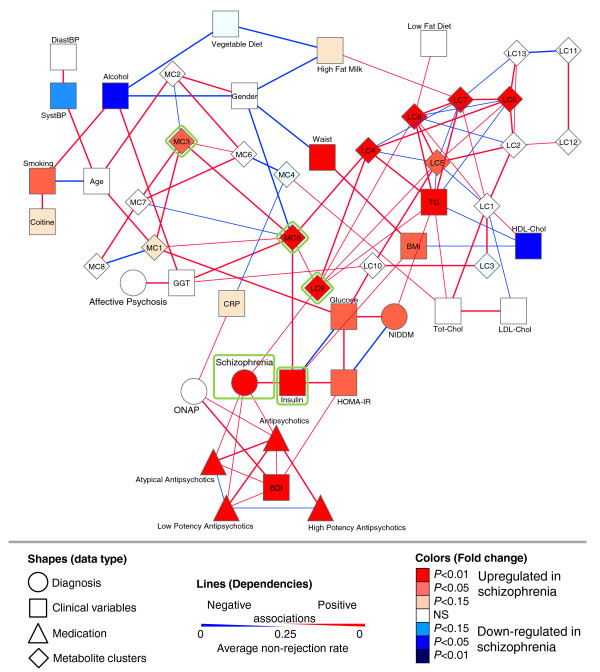
**Dependency network in schizophrenia and related psychoses**. The network was constructed from the diagnostic, clinical, antipsychotic medication use, and metabolite cluster data. Node shapes represent different types of variables and platforms, node color corresponds to significance and direction of regulation (schizophrenia versus controls), and line width is proportional to strength of dependency. The two metabolic variables directly linked with schizophrenia and two other metabolic network hubs are highlighted with green squares. The cutoff for the presence of an edge was set at *β *= 0.25 by the average non-rejection rate, that is, an edge in the graph was tested positive in 25% of the 500 samplings. Abbreviations: BDI, Beck Depression Inventory [26]; BMI, body mass index; Chol, cholesterol; CRP, C-reactive protein; DiastBP, diastolic blood pressure; GGT, gamma-glutamyltransferase; HDL, high-density lipoprotein; HOMA-IR, homeostatic model assessment index; LDL, low-density lipoprotein; NIDDM, non-insulin-dependent diabetes mellitus; SystBP, systolic blood pressure; TG, total triglycerides; Tot, total.

The linear mixed model analysis suggests that the dependencies of different metabolite classes and related metabolic phenotypes among themselves and with the specific diagnostic groups are likely complex. We hypothesized that a network approach may help elucidate these dependencies to a greater depth. In addition to diagnostic groups, which included also type 2 diabetes (non-insulin-dependent diabetes mellitus (NIDDM)) and the metabolite clusters, we selected 27 other environmental and phenotypic variables related to antipsychotic medication use, diet and lifestyle, metabolic phenotypes (for example, body mass index, insulin, glucose, HDL-cholesterol, total TG), and other biochemical measures, such as CRP and gamma-glutamyltransferase (GGT). The undirected Gaussian graphical Markov model was applied to estimate partial correlations between the variables (Figure 2).

In addition to variables related to antipsychotic use, schizophrenia was associated with two metabolic variables, lipid cluster LC9 and fasting serum insulin (Insulin in Figure 2). Insulin was further associated with related metabolic variables such as homeostatic model assessment (HOMA in Figure 2) index and glucose, while LC9 was associated with other TG-containing clusters as well as with total triglycerides. Both insulin and LC9 were associated with metabolite cluster MC5, which was directly linked to MC3. Neither the ONAP nor the affective psychosis group was directly associated with the specific metabolic clusters. ONAP was associated with the inflammatory marker CRP and with depressive symptoms. Affective psychosis was directly associated with the liver marker gamma-glutamyltransferase, which not surprisingly was associated with alcohol use.

### Feasibility of metabolic profile in assisting schizophrenia diagnosis

We reasoned that due to their independent association with schizophrenia, insulin as well as specific other metabolite clusters reflect the disease process itself, and may thus help discriminate schizophrenia from other psychoses. To assess the feasibility of diagnosis, we selected insulin as well as the top-ranking metabolites from three clusters of most interest based on the network structure in Figure 2: triglyceride TG(18:1/18:0/18:1) (LC9), isoleucine (MC3), and proline (MC5). Only the three psychotic groups were included in the analysis, without the controls, and the comparisons were made between the schizophrenia versus the pooled ONAP and affective psychosis groups.

**Figure 3:**
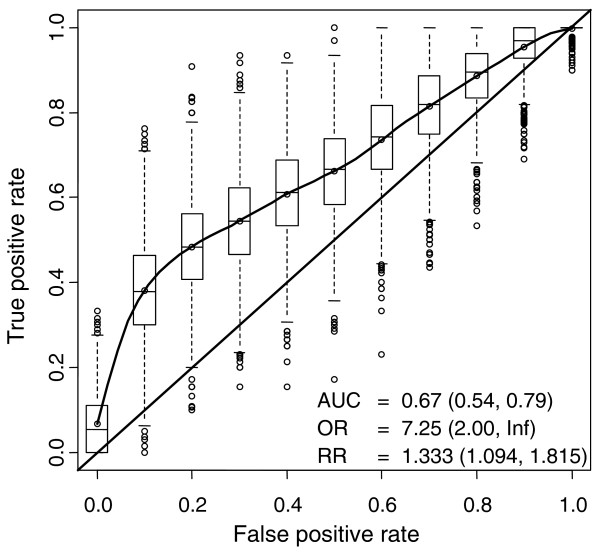
**Feasibility of diagnosing schizophrenia among different psychoses, based on proline and TG(18:1/18:0/18:1) concentrations**. The characteristics of the model (AUC, OR, RR) independently tested in one-third of the sample are shown as mean values (5th, 95th percentiles), based on 2,000 cross-validation runs. Abbreviations: AUC, area under the receiver operating characteristic (ROC) curve; OR, odds ratio; RR, relative risk.

The best model derived from logistic regression analysis was obtained by combining proline and TG(18:1/18:0/18:1). This combination was selected in 53% of 10,000 cross-validation runs. Other strongly performing models were proline alone (25%) and combined insulin and proline (13%). Figure 3 shows the summary of the combined proline and TG(18:1/18:0/18:1) diagnostic model, based on independently tested data taken from 2,000 samplings.

## Discussion

Our findings, based on a highly phenotypically detailed general population sample of different psychoses, independently associate specific metabolic phenotypes, as measured by metabolomics, with schizophrenia. It is known that schizophrenia is associated with elevated fasting total triglycerides and insulin resistance [39], but this metabolic abnormality has usually been attributed to antipsychotic drug-specific side effects [40]. The strongest association with schizophrenia based on network analysis as well as linear mixed models was with the lipid cluster LC9, which contains saturated and longer chain triglycerides. In a recent lipidomic study of different lipoprotein fractions in subjects with varying degrees of insulin resistance, we found that the lipids found in LC9 are abundant in liver-produced very low density lipoprotein particles and are associated with insulin resistance [41]. In agreement with this, schizophrenia patients in the present study were insulin resistant and had elevated fasting serum insulin levels. Together, our data indicate that schizophrenia, independent of antipsychotic medication and metabolic comorbidity, is characterized by insulin resistance, and consequently enhanced hepatic very low density lipoprotein production [42] and thus elevated serum concentrations of specific triglycerides. This is consistent with findings from an earlier study that demonstrated that antipsychotic medication-naïve patients with schizophrenia display hepatic insulin resistance independent of intra-abdominal fat mass or other known factors associated with hepatic insulin resistance [43].

The possible pathogenic relevance of our findings is supported by recent studies showing that abnormal insulin secretion and response [44-47] and abnormal glucose tolerance and risk of diabetes [48] are found already in drug-naïve first-episode patients with schizophrenia. In line with this, the insulinotropic [49,50] BCAAs from the metabolic cluster MC3 were also elevated and specifically associated with schizophrenia. In the context of psychoses, BCAAs are not only important due to their role in stimulating insulin secretion, but also since they compete with aromatic amino acids for transport across the blood-brain barrier [51]. Their increase may thus lead to concentration decreases of neurotransmitters derived from the aromatic amino acids in the brain, specifically catecholamines from tyrosine and phenylalanine (MC3) and serotonin from tryptophan (MC5). However, the effect of BCAA-induced dopamine or serotonin depletion in the brain on schizophrenia-related cognitive performance is currently controversial [52,53]. Another potential mechanism linking schizophrenia and long-term hyperinsulinemia is dysregulation of insulin-receptor-mediated signaling, which has a role in learning and memory as well as in regionally specific glucose metabolism in the brain [54].

The metabolic cluster MC5, which included proline and glutamate, was strongly associated with schizophrenia. Glutamate has been hypothesized to play an important role in schizophrenia [55]. Our data show that serum glutamate is elevated in all psychoses compared to controls (Figure 1), supporting the view that glutamate-related metabolic abnormalities may reflect a common pathway across different psychoses [56]. However, one should also note that the dependency of glutamate concentrations between the brain and blood is weak and complex due to restricted and tightly controlled passage of glutamate across the blood-brain barrier [57].

Upregulation of serum proline was specific to schizophrenia. There is evidence from genetics that polymorphisms in the *PRODH *gene, encoding proline oxidase, which is located at 22q11, are associated with schizophrenia risk [58,59] and that the related hyperprolinemia negatively associates with cognitive performance [60]. In particular, functional variants in the *PRODH *gene that result in reduction of proline oxidase activity and hyperprolinemia are associated with increased risk of schizophrenia and changes in fronto-striatal structure and function [59,61]. Interestingly, schizophrenia is linked to the same copy number variants spanning the 22q11 region including *PRODH *as autism and other childhood developmental disorders, whereas bipolar disorder is not [62]. Furthermore, recent functional studies suggest that microdeletions on human chromosome 22 (22q11.2) lead to impaired long-range synchrony of neuronal activity and may thus be an important component of the pathophysiology of schizophrenia [63].

Having a population-based sample with carefully matched controls was a definite strength of the study. The Psychoses in Finland study has been characterized as 'arguably the most thorough study ever undertaken on the prevalence of psychotic disorders' [64]. In addition to the careful screening and assessment of psychotic disorders, the assessment of health and lifestyle in the Health 2000 survey was comprehensive. Diabetes and metabolic syndrome had been carefully diagnosed [24,39] and their effects had been controlled for in the analyses. Notably, most antipsychotics used by patients were first-generation antipsychotics, which are less associated with diabetes compared to second-generation antipsychotics [65]. However, the sample was relatively old and the mean duration of illness among subjects with psychotic disorders had been long. Although we controlled for the effects of current lifestyle, all the long-term effects of antipsychotic medication and lifestyle-related factors, like smoking, nutrition and exercise, may not have been captured. Nevertheless, studies on drug-naïve first-episode patients already find impaired glucose tolerance, elevated insulin and metabolic abnormalities [43-48] that are not related to poor health habits [48]. Longitudinal research in prodromal and early psychosis is needed to further elucidate the role of the identified metabolomic changes in psychotic disorders.

## Conclusions

Our study suggests that proline-related metabolic abnormalities and insulin secretion-related changes (BCAAs, insulin, triglycerides) reflect two different disease-related pathways. This is further supported by the fact that the best candidate diagnostic model separating schizophrenia from other psychoses is obtained by combining the selected metabolites from each of the two pathways. We believe metabolomics, which is sensitive to both genetic and environmental variation, will be a powerful tool to further investigate susceptibility to psychotic disorders, their clinical course, and treatment responses.

## Abbreviations

BCAA: branched chain amino acid; BDI: Beck Depression Inventory; CRP: C-reactive protein; DSM: *Diagnostic and Statistical Manual of Mental Disorders*; GC × GC-TOFMS: two-dimensional gas chromatography coupled to time-of-flight mass spectrometry; HDL: high-density lipoprotein; LDL: low-density lipoprotein; MS: mass spectrometry; ONAP: other non-affective psychoses; PC: phosphatidylcholine, PE: phosphatidylethanolamine; SCID: Structured Clinical Interview for DSM-IV; TG: triglyceride; UPLC: ultra-performance liquid chromatography.

## Competing interests

The authors declare that they have no competing interests.

## Authors' contributions

JT, MSA, MO, and JS performed the statistical analysis. TSL, IM, and TH carried out metabolomic analyses. JL and JP participated in the study design. SES, SIS, JP, and JS researched the primary clinical data. MO and JS conceived of the study, and participated in its design and coordination and drafted the manuscript. All authors read and approved the final manuscript.

## Supplementary Material

Additional file 1 **Supplementary Table 1**. Linear mixed models, with diagnostic categories, current antipsychotic medication use, diet, metabolic comorbidity (obesity or metabolic syndrome) and fasting time as explanatory variables. People with type 2 diabetes and their matched controls were excluded from the analysis. Click here for file
