# Supplementary material for: Metabolome in schizophrenia and other psychotic disorders: a general population-based study
Source: Genome Med. 2011 Mar 23;3(3):19. doi: 10.1186/gm233 (PMC3092104; doi:10.1186/gm233)
Supplement: Additional file 1 — Supplementary Table 1. Linear mixed models, with diagnostic categories, current antipsychotic medication use, diet, metabolic comorbidity (obesity or metabolic syndrome) and fasting time as explanatory variables. People with type 2 diabetes and their matched controls were excluded from the analysis. [file gm233-S1.PDF]

Supplementary Table 1. Linear mixed models, with diagnostic categories, current antipsychotic medication use, diet, metabolic comorbidity (having either obesity or metabolic syndrome) and fasting time as explanatory variables. Persons with type 2 diabetes and their matched controls were excluded from the analysis.

Cluster name	Significant predictors
**C1**	none
**LC2**	none
**LC3**	none
**LC4**	schizophrenia (↑, *t*=4.21, *P*<0.0001), daily use of cheese with high fat content (↑, *t*=2.53, P= 0.013), metabolic comorbidity (↑, *t*=4.88, *P*<0.0001)
**LC5**	metabolic comorbidity (↑, *t*=2.69, *P*=0.009)
**LC6**	schizophrenia (↑, *t*=2.30, *P*=0.023), daily use of cheese with high fat content (↑, *t*=2.24, P=0.028), metabolic comorbidity (↑, *t*=3.47, *P*=0.0008)
**LC7**	schizophrenia (↑, *t*=3.71, *P*=0.0003), daily use of cheese with high fat content (↑, *t*=2.38, P=0.019), use of milk with high fat content (↑, *t*=2.01, P=0.047), metabolic comorbidity (↑, *t*=7.13, *P*<0.0001)
**LC8**	schizophrenia (↑, *t*=3.62, *P*=0.0005), metabolic comorbidity (↑, *t*=5.93, *P*<0.0001)
**LC9**	schizophrenia (↑, *t*=4.64, *P*<0.0001), daily use of cheese with high fat content (↑, *t*=2.79, *P*=0.006), use of milk with high fat content (↑, *t*=2.15, *P*=0.034), metabolic comorbidity (↑, *t*=5.35, *P*<0.0001)
**LC10**	metabolic comorbidity (↑, *t*=5.04, *P*<0.0001)
**LC11**	ONAP (↑, *t*=2.03, *P*=0.045), use of vegetable oils (↓, *t*=-3.58, *P*=0.0005), fasting time (↓, *t*=-2.39, *P*=0.019)
**LC12**	ONAP (↑, *t*=2.96, *P*=0.004), use of vegetable oils (↓, *t*=-3.24, *P*=0.002), fasting time (↓, *t*=-2.50, *P*=0.014)
**LC13**	Daily use of vegetables (↑, *t*=2.11, *P*=0.038)
**MC1**	schizophrenia (↑, *t*=2.01, *P*=0.048)
**MC2**	affective psychosis (↓, *t*=-2.59, *P*=0.011), antipsychotic use (↑, *t*=2.49, *P*=0.014). Note: association with schizophrenia (*t*=-1.98, *P*=0.0504)
**MC3**	schizophrenia (↑, *t*=2.15, *P*=0.034)
**MC4**	antipsychotic use (↓, *t*=2.26, *P*=0.026), use of vegetable oils (↑, *t*=2.54, *P*=0.013)
**MC5**	metabolic comorbidity (↑, *t*=3.77, *P*=0.0003), fasting time (↓, *t*=2.01, *P*=0.047). Note: association with schizophrenia (*t*=1.53, *P*=0.13)
**MC6**	none
**MC7**	none
**MC8**	none
